# A Genome-Wide Association Study for Nutritional Indices in *Drosophila*

**DOI:** 10.1534/g3.114.016477

**Published:** 2015-01-12

**Authors:** Robert L. Unckless, Susan M. Rottschaefer, Brian P. Lazzaro

**Affiliations:** Department of Entomology, Cornell University, Ithaca, New York 14853

**Keywords:** *Drosophila*, DGRP, glycogen, glucose, triglyceride, protein, weight

## Abstract

Individuals are genetically variable for the way in which they process nutrients and in the effects of dietary content on reproductive success, immunity, and development. Here, we surveyed genetic variation for nutrient stores (glucose, glycogen, glycerol, protein, triglycerides, and wet weight) in the *Drosophila* Genetic Reference Panel (DGRP) after rearing the flies on either a low-glucose or high-glucose diet. We found significant genetic variation for these nutritional phenotypes and identified candidate genes that underlie that variation using genome-wide associations. In addition, we found several significant correlations between the nutritional phenotypes measured in this study and other previously published phenotypes, such as starvation stress resistance, oxidative stress sensitivity, and endoplasmic reticulum stress, which reinforce the notion that these lines can be used to robustly measure related phenotypes across distinct laboratories.

The quality of dietary nutrition and the assimilation of dietary nutrients have significant influence on many traits, including lifespan (Piper *et al.* 2005; Piper and Partridge 2007; Skorupa *et al.* 2008), development (Layalle *et al.* 2005), reproduction (Fricke *et al.* 2008), and immunity (Ayres and Schneider 2009; Fellous and Lazzaro 2010; Vass and Nappi 1998). Resources such as the *Drosophila* Genetic Reference Panel (DGRP) provide a practical means of using natural genetic variation to both untangle the genetic basis of complex traits and understand the intersection of selection and genetics in the maintenance of that variation (Mackay *et al.* 2012). The DGRP is a set of approximately 200 *D. melanogaster* genetic lines that have been genome-sequenced and are available to the community for the mapping of complex genetic traits. Here, we present the results of a genome-wide scan for SNPs associated with several nutritional indices measured after rearing on either a low -glucose (1 glucose: 2 yeast) diet or a high-glucose (2 glucose: 1 yeast) diet. We found significant genetic variation for all traits (total soluble protein, glucose, glycogen, free glycerol, triglycerides, and wet weight) and were able to map underlying genes. We additionally note correlations between our nutritional indices and several previously published DGRP phenotypes (Mackay *et al.* 2012; Jordan *et al.* 2012; Ayroles *et al.* 2009; Chow *et al.* 2013b).

## Materials and Methods

### *Drosophila* stocks and husbandry

We assayed nutritional indices in the DGRP (Mackay *et al.* 2012), a collection of approximately 200 inbred lines of *Drosophila melanogaster* derived from wild-caught females (2003, Raleigh, NC). Our study utilized 172 of these lines, although not every line was available for every day of the experiment.

Before measuring any phenotypes, each line was reared for at least three generations on two diets that varied in glucose content. The low-glucose diet consisted of 5% weight by volume brewer’s yeast (MP Biomedicals, Santa Ana, CA), 2.5% glucose (Sigma-Aldrich, St. Louis, MO), and 1% *Drosophila* agar (Genesee Scientific, San Diego, CA) supplemented with 800 mg/L methyl paraben (Sigma-Aldrich), and 6 mg/L carbendazim (Sigma-Aldrich). The high-glucose diet was exactly the same but consisted of 10% glucose.

### Nutrient indices in the DGRP

We assayed nutritional indices in pools of 10 adult males from each line aged 3–6 days after eclosion. We measured free glucose, glycogen stores, total triglycerides, free glycerol, and soluble protein in groups of 10 male flies, with three biological replicates of rearing on each diet. Each group of flies was weighed using a MX5 microbalance (Mettler-Toledo, Columbus, OH) and then homogenized in 200 μL buffer (10 mM Tris, 1 mM EDTA, pH 8.0 with 0.1% v/v Triton-X-100) using lysing matrix D (MP Biomedicals, Santa Ana, CA) on a FastPrep-24 homogenizer (MP Biomedicals). We immediately froze 50 μL of the homogenate to be used for the total protein assay and incubated the remaining 150 μL at 72° for 20 min to denature enzymes naturally present in the homogenate. Each nutritional index was assayed using modifications of commercially available kits (see Unckless *et al.* unpublished data; Ridley *et al.* 2012): glucose with the oxidase kit (GAGO-20; Sigma-Aldrich); glycogen using the glucose kit and amyloglucosidase from *Aspergillus niger* (A7420; Sigma-Aldrich) in 10 mM acetate buffer at pH 4.6; free glycerol and triglycerides using reagent kits F6428 and T2449, respectively (Sigma-Aldrich); and soluble protein with the DC Protein Assay (BIO-RAD, Hercules, CA).

### Data analysis

Before genome-wide association mapping, we estimated line means for each nutritional index using abundance of metabolite per mg of fly. The model used was:

$$Y_{ijklmn}=m+\mathrm{Wol}b_{i}+\mathrm{Die}t_{j}+\mathrm{Lin}e_{k}\left(\mathrm{Wol}b_{i}\right)+\mathrm{Bloc}k_{n}\left(\mathrm{Die}t_{j}\right)+\mathrm{Die}t_{j}\mathrm{\times Lin}e_{k}\left(\mathrm{Wol}b_{i}\right)+e_{ijklmn}$$

where Y is the estimated mass (μg) per fly of each nutrient divided by the mass of the flies measured in mg (except, obviously, in the case where wet mass is itself the response variable). Wolb*_i_* (*i* = 1,2) indicates endosymbiotic bacterium *Wolbachia pipientis* infection status, Diet*_j_* (*j* = 1,2) indicates rearing diet, Block*_n_*(Diet*_j_*) (*n* = 1,3) differentiates among the three replicate blocks within each diet, Line*_k_*(Wolb*_i_*) (*k* = 1,2,…,172) tests the influence of inbred line on nutritional index nested within *Wolbachia* infection status (52.2% of lines were infected), and the Diet*_j_* × Line*_k_*(Wolb*_i_*) interaction term tests whether inbred lines differ in their responsiveness to the two diets. All factors were considered fixed. All models were run in SAS 9.3 (Cary, NC) using the “GLM” procedure and least squares means were extracted. For modeling on each diet individually, the model used was: $Y_{ijkl}=m+\mathrm{Wol}b_{i}+\mathrm{Lin}e_{k}\left(\mathrm{Wol}b_{i}\right)+\mathrm{Bloc}k_{l}+e_{ikl}$.

We also obtained a more holistic view of fly metabolic status by performing a principal component analysis on the collective set of nutritional measures, excluding wet weight, because that is implicitly contained in mass-scaled measures of individual nutrients. This allowed us to distill the higher-order interactions of our nutritional phenotypes into several one-dimensional components. Line estimates for each nutritional principal component were determined using the *prcomp* function in R (R Core Team 2012) with *tol* = 0.1 and unit variance scaling turned on. This analysis was completed with flies reared on the two diets considered separately.

### Genome-wide association mapping

The set of SNPs for genome-wide association mapping was described in Huang *et al.* (2014) and consists of only SNPs with minor alleles present in at least four of the lines (MAF >2%; 2,415,518 total SNPs). For genome-wide associations, we formatted this SNP set for PLINK-assessed (Purcell *et al.* 2007) associations between SNP and line estimates from the above models using the “–assoc” flag to perform associations and the “–qt-means” flag for estimates of effect size. PLINK uses an ordinary least squares model for each SNP. These analyses were performed for the high-glucose diet, low-glucose diet, and when data from both diets were pooled. We used a nominal *P* value threshold of *P* < 10^−6^ for declaring SNPs to be significantly associated with trait variation but relaxed this to *P* < 10^−4^ for gene ontology enrichment analysis (see below).

### GO term analysis

We performed Gene Ontology (GO) analysis corrected for gene size using GOWINDA (Kofler and Schlötterer 2012) to test for the enrichment of particular functional groups in genes bearing SNPs associated with variation in phenotypic traits. Significantly associated SNPs (*P* < 10^−4^) for each treatment (low glucose, high glucose, main effect) were used as the query set with a background SNP set consisting of all remaining SNPs used in the genome-wide mapping. We used this relaxed *P* value threshold to increase the number of significant SNPs in this analysis. GO slim (Adams *et al.* 2000) terms were used to reduce redundancy in GO categories. GOWINDA was run using *gene* mode, including all SNPs within 1000 bp of a gene, a minimum gene number of 5, and with 100,000 simulations. We report all GO terms with a nominal *P* < 0.1.

### Phenotypic correlations with other traits

We examined correlations among our measured traits, and between our nutritional phenotypes and independent traits that have been measured in the DGRP lines by other research groups. Correlation analyses were performed in R (R Core Team 2012) using our line mean estimates, and we report both correlation coefficient and *P* value. For significantly correlated traits, we queried whether a single gene or a few genes might drive the correlation by determining whether the same SNPs were significantly associated with variation in both traits with a relaxed *P* value threshold of 10^−5^.

## Results

### Genetic and environmental variation for nutritional status across the DGRP

ANOVA for each nutritional index (both pooled across diets and on each diet individually) is presented in Supporting Information, Table S1. When the data from each diet are analyzed separately, all nutritional indexes showed a significant (or nearly significant) line effect except soluble protein after rearing on the low-glucose diet and triglycerides after rearing on the high-glucose diet (Table S1b), indicating that most traits are genetically variable. When the data from both diets were pooled, all nutritional indices except free triglycerides and glycogen showed a significant effect of rearing diet, with glucose, glycerol, and triglycerides occurring at higher levels in flies reared on the high-glucose diet, whereas glycogen, soluble protein, and total wet mass were lower in flies reared on the high-glucose diet. All nutritional indices showed a significant effect of line. Only wet weight showed a significant interaction between line and diet (Table S1a). In addition, total soluble protein showed a significant effect of *Wolbachia* infection status (*P =* 0.047). All phenotypic values are presented in Table S2.

### Principal components of nutritional indices

We considered that our nutritional indices might give more information about the metabolic status of the fly when considered in aggregate, so we used a principle component (PC) analysis to extract the top five PCs from the full nutritional data set. The top five principal components summarizing the NIs on each diet each explain 12–31% of the total in nutritional state, with loadings of each NI given in Table S3. Principal component loadings show variation in both sign and magnitude of contribution from each NI, suggesting they capture complex integrations of the nutritional indices to reflect overall metabolic state.

### Phenotypic correlations with other traits

We measured correlations between our nutritional phenotypes and several other traits that have been measured in the DGRP and for which the data are publically available (starvation stress resistance, chill coma recovery, startle response, oxidative stress response, endoplasmic reticulum stress) (Mackay *et al.* 2012; Jordan *et al.* 2012; Chow *et al.* 2013b). Table 1 contains the correlation coefficient and *P* value for each trait combination. Note that for all nutritional indices, we present correlations between other phenotypes and line means estimated when data from both diets were pooled. We did not perform principal components analysis on this pooled data; however, diet-specific principal components were used for the analysis.

**Table 1:** Correlations between our nutritional indices and traits previously measured by other groups: principal components

Phenotype	Starvation Stress Resistance	Chill Coma	Startle Response	Paraquat	MSB	ER Hazard Ratio	ER T_50_	Male Reproductive Fitness*^d^*	Lifespan*^d^*	Male Aggression*^d^*	Mating*^d^*	Ethanol Tolerance*^d^*
Source	1	1	1	2	2	3	3	4	4	4	4	4
Glucose	**0.246***^b^*	**−0.245***^b^*	**0.309***^b^*	0.069	0.043	−0.057	0.076	−0.134	−0.073	0.303	−0.043	−0.048
Glycogen	**0.307***^c^*	*−0.168**^a^*	**0.249***^b^*	*0.186**^a^*	*0.197**^a^*	0.099	**0.279***^b^*	0.163	0.183	0.224	0.212	0.041
Glycerol	0.079	0.005	0.008	0.012	−0.013	−0.020	**0.286***^b^*	−0.050	−0.227	−0.235	−0.175	−0.225
Triglycerides	−0.071	−0.081	0.003	−0.045	−0.107	−0.038	*-0.236**^a^*	0.067	0.042	0.245	0.244	−0.129
Protein	−0.113	*−0.177**^a^*	−0.093	*−0.183**^a^*	−0.037	−0.157	−0.178	*−0.428**^a^*	0.057	−0.285	−0.030	0.178
Wet weight	**0.241***^b^*	**−0.238***^b^*	0.030	*0.180**^a^*	*0.191**^a^*	−0.175	0.165	−0.146	0.166	*−0.358**^a^*	0.066	0.231
LGD PC1	**−0.261***^b^*	−0.090	−0.143	−0.132	−0.136	−0.058	**−0.305***^b^*	0.148	0.066	0.111	0.115	0.124
LGD PC2	*0.207**^a^*	−0.052	*0.177**^a^*	0.152	0.114	0.072	0.037	0.184	0.113	0.242	0.288	0.050
LGD PC3	**0.226***^b^*	**−0.318***^c^*	0.013	0.152	0.090	*0.196**^a^*	**0.368***^c^*	0.214	0.058	0.021	−0.280	0.155
LGD PC4	−0.098	−0.110	0	−0.020	−0.136	0.042	0.049	0.217	**−0.462***^b^*	−0.003	0.013	0.105
LGD PC5	0.087	−0.070	0.093	0.016	−0.065	−0.052	−0.054	**0.459***^b^*	−0.027	0.158	−0.130	0.021
HGD PC1	**0.309***^c^*	**−0.248***^b^*	**0.279***^c^*	0.096	0.051	−0.016	0.186	−0.015	0.086	0.338	0.104	−0.112
HGD PC2	−0.003	−0.019	−0.102	−0.093	−0.038	−0.043	0.091	−0.154	0.006	−0.281	−0196	0.120
HGD PC3	−0.035	−0.035	−0.068	−0.070	−0.079	0.046	*−0.251**^a^*	−0.232	0.292	0.166	−0.042	0.025
HGD PC4	0.028	−0.087	−0.094	0.026	−0.096	−0.044	0.016	−0.008	−0.026	−0.043	−0.082	**−0.737***^c^*
HGD PC5	0.135	−0.116	−0.001	0.141	0.096	0.163	*0.216**^a^*	0.059	0.102	0.144	0.253	−0.086

All nutritional indices (protein, glucose, etc.) are values found when data from both diets were pooled. For correlation coefficients, cells in italics are *P* < 0.05 and cells in bold are *P* < 0.01. HGD, high-glucose diet; LGD, low -glucose diet; 1, Mackay *et al.* 2012; 2, Jordan *et al.* 2012; 3, Chow, Wolfner, and Clark 2013b; 4, Ayroles *et al.* 2009.

a *P* < 0.05

b *P* < 0.01.

c *P* < 0.001.

d Correlations performed with only 40 DGRP lines.

Several interesting correlations are evident. In particular, starvation stress resistance as measured by Mackay *et al.* (2012) is correlated with several metabolic principal components and is positively correlated with wet weight (*P =* 0.005) and with levels of glucose (*P =* 0.004) and glycogen (*P* < 0.001). Chill coma recovery, also measured by Mackay *et al.* (2012), is correlated with two metabolic principal components as well as with wet weight (*P =* 0.005), levels of glucose (*P* = 0.004), glycogen (*P =* 0.048), and protein (*P =* 0.038). Startle response (Mackay *et al.* 2012) is correlated with two metabolic principal components and with glucose (*P* < 0.001) and triglyceride (*P =* 0.003) levels. Sensitivity to oxidative stress, induced by either paraquat or menadione sodium bisulfate (MSB) (Jordan *et al.* 2012), was positively correlated with glycogen stores (*P =* 0.029 and *P =* 0.021, respectively) and wet weight (*P =* 0.035 and *P =* 0.025, respectively). Sensitivity to paraquat was also negatively correlated with soluble protein (*P =* 0.032). Interestingly, several nutritional indices were significantly correlated with time to 50% mortality after endoplasmic reticulum stress (ER T_50_) (Chow *et al.* 2013b), including glycogen stores (*P =* 0.005), glycerol level (*P =* 0.004), total triglycerides (*P =* 0.020), as well as PC1 and PC3 on the low-glucose diet and PC1 on the high-glucose diet.

Phenotypic values for male reproductive fitness, male aggression, lifespan, and ethanol tolerance were also reported for a smaller set of 40 DGRP lines (Ayroles *et al.* 2009). With only 40 lines, we have less power to find correlations with these data, although we do still detect some significant correlations. Male reproductive fitness (proportion of offspring sired during competition for matings with males from a marked stock) is negatively correlated with our measure of soluble protein (*P =* 0.015) and positively correlated with low-glucose diet PC5. Lifespan is positively correlated with low-glucose diet PC4. Surprisingly, male aggression as determined by Ayroles *et al.* was negatively correlated with our measure of wet weight (*P =* 0.044), where we might have naively expected larger flies to be more aggressive. Finally, ethanol tolerance is significantly positively correlated with high-glucose PC4.

### Genome-wide association results

SNPs that are significantly associated with variation in each nutritional phenotype (*P* < 10^−6^) are presented in Table 2 and Table 3. Overall, SNPs significantly associated with variation in our nutritional phenotypes are disproportionately found as nonsynonymous substitutions or in introns and UTRs, as opposed to synonymous substitutions or positions more than 1000 bp from known genes, relative to the distribution of all variants across the genome. For the nutritional indices, 33 out of 48 (69%) total significantly associated SNPs across phenotypes and diets are found in introns, UTRs, less than 1000 bp from an annotated gene, or as nonsynonymous SNPs. For principal components, this fraction is 17 of 24 (71%). In contrast, less than half of all SNPs meeting criteria for inclusion in this study are found in introns or UTRs, are less than 1000 bp from an annotated gene, or are nonsynonymous. This enrichment for putatively functional SNPs is significant (χ^2^ = 6.75, df = 1, *P* = 0.009 for nutritional indices; χ^2^ = 4.17, df = 1, *P* = 0.041 for principal components). For example, across the three mapping strategies (low glucose, high glucose and data pooled across diets), there were seven unique SNPs meeting our threshold for association with glucose levels. Of these, one was synonymous and one was not associated with any known gene. The remaining five mapped SNPs were intronic. For triglyceride levels, all four significantly associated SNPs were intronic. Each SNP that associates significantly with variation in a measured phenotype is given in Table 2, including significance level, estimated effect size, minor allele frequency, type of SNP, and gene functional categorization. No SNPs were significantly associated with more than one distinct nutritional phenotype, even when the significance threshold was relaxed to 10^−5^.

**Table 2:** SNPs significantly associated with variation in nutritional indices at *P* < 10^−6^

NI	Diet	SNP	*P*	Effect	MAF	Gene	FBgn	Type	Function	Reference
Glucose	HGD	3L.4811585	4.83E-07	−0.020	0.394	*Dhc64C*	FBgn0051025	Syn.	Cellularization	Papoulas *et al.* 2005
		3R.6404817	3.41E-07	−0.022	0.222	*hth*	FBgn0001235	Intron	Brain development	Nagao *et al.* 2000
		3R.23998828	9.64E-07	−0.021	0.307	CG34354	FBgn0085383	Intron	Nucleic acid binding	Tweedie *et al.* 2009
	LGD	NA	NA	NA	NA	NA	NA	NA	NA	NA
	Pooled	3R.6290881	8.25E-07	−0.023	0.075	NA	NA	NA	NA	NA
		3R.6404817	1.35E-07	−0.015	0.226	*hth*	FBgn0001235	Intron	Brain development	Nagao *et al.* 2000
		3R.6440408	9.67E-07	−0.016	0.176	*hth*	FBgn0001235	Intron	Brain development	Nagao *et al.* 2000
		3R.6446314	8.68E-07	−0.016	0.179	*hth*	FBgn0001235	Intron	Brain development	Nagao *et al.* 2000
		3R.6455818	3.57E-08	−0.018	0.154	*hth*	FBgn0001235	Intron	Brain development	Nagao *et al.* 2000
Glycerol	HGD	2R.18726642	5.41E-07	0.014	0.297	CG9825	FBgn0034783	Syn.	Transmembrane transport	Tweedie *et al.* 2009
	LGD	X.6541116	4.83E-07	0.013	0.224	*pig*	FBgn0029881	Intron	Small body	Tweedie *et al.* 2009
		X.6541138	2.35E-07	0.014	0.229	*pig*	FBgn0029881	Intron	Small body	Tweedie *et al.* 2009
		X.6541155	7.14E-07	0.013	0.229	*pig*	FBgn0029881	Intron	Small body	Tweedie *et al.* 2009
		X.6541215	2.24E-07	0.014	0.215	*pig*	FBgn0029881	Intron	Small body	Tweedie *et al.* 2009
	Pooled	2L.307423	1.16E-07	−0.016	0.141	*Plc21C*	FBgn0004611	Intron	Lipid catabolic process	Tweedie *et al.* 2009
		3L.11508784	7.70E-07	0.011	0.353	CG7512	FBgn0036168	Intron	Metal ion binding	Tweedie *et al.* 2009
		3R.14453686	1.54E-07	−0.010	0.482	*Qin*	FBgn0263974	Intron	Protein autoubiquination	Tweedie *et al.* 2009
Glycogen	HGD	2R.7673484	4.56E-07	−0.023	0.210	*ths*	FBgn0033652	Intron	Fibroblast growth factor binding	Itoh and Ornitz 2004
		2R.17598285	2.15E-07	0.021	0.328	CG30403	FBgn0050403	Intron	DNA binding	Tweedie *et al.* 2009
		2R.17598285	2.14E-07	0.021	0.338	CG30403	FBgn0050403	Intron	DNA binding	Tweedie *et al.* 2009
	LGD	2L.8316116	4.93E-07	−0.022	0.073	CG7806	FBgn0032018	Syn.	Transmembrane transport	Tweedie *et al.* 2009
	Pooled	2L.11397732	4.66E-07	−0.023	0.217	*NA*	NA	NA	NA	NA
Mean weight	HGD	NA	NA	NA	NA	*NA*	NA	NA	NA	NA
	LGD	2L.3261343	6.08E-07	56.1	0.321	*NA*	NA	NA	NA	NA
		2L.3271697	7.11E-07	53.6	0.420	CG3347	FBgn0031513	3′ UTR	Zinc ion binding	Tweedie *et al.* 2009
		3R.25948794	7.31E-07	60.9	0.239	CG45072	FBgn0266442	Nonsyn.	Unknown	NA
		3R.25948812	8.9E-07	60.1	0.245	CG45072	FBgn0266442	5′ UTR	Unknown	NA
		3R.25952830	4.90E-09	84.3	0.150	*Ppi1*	FBgn0051025	Nonsyn.	Protein phosphatase inhibitor	Bennett *et al.* 2006
		3R.25952966	1.37E-08	83.2	0.143	*Ppi1*	FBgn0051025	Syn.	Protein phosphatase inhibitor	Bennett *et al.* 2006
		3R.25953010	9.67E-09	78.5	0.169	*Ppi1*	FBgn0051025	Nonsyn.	Protein phosphatase inhibitor	Bennett *et al.* 2006
		3R.25953104	5.00E-09	84.1	0.152	*Ppi1*	FBgn0051025	Syn.	Protein phosphatase inhibitor	Bennett *et al.* 2006
		3R.25953203	2.30E-08	76.4	0.155	*Ppi1*	FBgn0051025	Syn.	Protein phosphatase inhibitor	Bennett *et al.* 2006
		3R.25953305	4.36E-08	76.9	0.161	*Ppi1*	FBgn0051025	Syn.	Protein phosphatase inhibitor	Bennett *et al.* 2006
	Pooled	3R.25952830	2.90E-08	73.9	0.148	*Ppi1*	FBgn0051025	Nonsyn.	Protein phosphatase inhibitor	Bennett *et al.* 2006
		3R.25952966	1.21E-07	71.5	0.141	*Ppi1*	FBgn0051025	Syn.	Protein phosphatase inhibitor	Bennett *et al.* 2006
		3R.25953010	3.49E-07	65.1	0.168	*Ppi1*	FBgn0051025	Nonsyn.	Protein phosphatase inhibitor	Bennett *et al.* 2006
		3R.25953104	5.44E-08	72.3	0.150	*Ppi1*	FBgn0051025	Syn.	Protein phosphatase inhibitor	Bennett *et al.* 2006
		X.5877626	5.01E-07	−48.3	0.423	*Grip*	FBgn0029830	Intron	Glutamate receptor binding; muscle attachment	Swan *et al.* 2004
Protein	HGD	NA	NA	NA	NA	NA	NA	NA	NA	NA
	LGD	2L.1888490	6.48E-07	−0.016	0.298	*CG7337*	FBgn0031374	Intron	Quinonprotein alcohol dehydrogenase activity	Tweedie *et al.* 2009
		2L.7008495	2.62E-07	−0.021	0.149	*uif*	FBgn0031879	5′ UTR	Notch binding	Tweedie *et al.* 2009
		3R.4370437	7.68E-07	−0.020	0.145	*NA*	NA	NA	NA	NA
		3R.15771872	7.28E-07	−0.018	0.216	*Hs6st*	FBgn0038755	Intron	Sulfotransferase	Ghabrial *et al.* 2003
Protein (cont.)		3R.18325276	2.41E-07	−0.023	0.140	*oa2*	FBgn0038980	Intron	Octopamine receptor activity	Balfanz *et al.* 2005
	Pooled	2R.17648180	6.69E-07	−0.013	0.512	*NA*	NA	NA	NA	NA
		3L.6131752	5.82E-07	0.012	0.482	Cpr65Av	FBgn0052405	Down (571)	Insect cuticle protein	Karouzou *et al.* 2007
						Lcp65Ae	FBgn0020640	Up (534)	Insect cuticle protein	Karouzou *et al.* 2007
Triglycerides	HGD	X.20411124	3.85E-07	−0.020	0.222	*RunxB*	FBgn0259162	Intron	Cellular process	Boutros *et al.* 2004
	LGD	2L.4905518	8.54E-07	0.025	0.353	CG2837	FBgn0031646	Intron	Unknown	NA
		2R.15064256	9.66E-07	−0.025	0.331	CG10737	FBgn0034420	Intron	Intracellular signal transduction	Tweedie *et al.* 2009
		X.5445429	4.50E-07	0.033	0.188	*Vsx2*	FBgn0263512	Intron	DNA binding	Tweedie *et al.* 2009
	Pooled	NA	NA	NA	NA	NA	NA	NA	NA	NA

Effect, effect size of minor allele; SNPs labeled NA are not within 1000 bp of an annotated gene. Lines with all NAs indicate no SNPs met significance threshold; MAF, minor allele frequency.

**Table 3:** SNPs significantly associated with variation in principal components of nutritional indices at P < 10−6

PC	Diet	SNP	*P*	Effect	MAF	Gene	FBgn	Type	Function	Reference
PC1	HGD	2L.15990382	9.79E-07	−0.988	*0.465*	*CR43412*	FBgn0263331	Down (435)	Nonprotein coding	Tweedie *et al.* 2009
		X.16918901	9.24E-07	1.046	*0.300*	*CG43997*	FBgn0264739	Down (923)	Unknown	NA
PC2	HGD	3L.1235270	7.76E-08	−1.015	*0.309*	*CG33966*	FBgn0053966	3′ UTR	Vitamin E binding	Tweedie *et al.* 2009
		3L.1235273	8.26E-07	−0.927	*0.324*	*CG33966*	FBgn0053966	3′ UTR	Vitamin E binding	Tweedie *et al.* 2009
		3L.2644168	9.74E-07	0.974	*0.264*	*CG14949*	FBgn0035358	Up (744)	Unknown	NA
		3L.17498584	9.88E-07	0.970	*0.270*	*Oatp74D*	FBgn0036732	Intron	Organic anion transport	Tweedie *et al.* 2009
PC3	HGD	X.20411124	5.38E-07	−0.977	*0.222*	*RunxB*	FBgn0259162	Intron	DNA binding	Boutros *et al.* 2004
PC4	HGD	3R.14205878	2.25E-07	−0.976	*0.178*	*CG7675*	FBgn0038610	Intron	Glucose/ribitol reductase	Tweedie *et al.* 2009
		3R.14206166	2.70E-08	−1.016	*0.200*	*CG7675*	FBgn0038610	Intron	Glucose/ribitol reductase	Tweedie *et al.* 2009
		3R.14206170	2.34E-07	−0.924	*0.211*	*CG7675*	FBgn0038610	Intron	Glucose/ribitol reductase	Tweedie *et al.* 2009
		X.8316242	5.06E-07	−1.630	*0.080*	*NA*	NA	NA	NA	NA
PC5	HGD	2L.18537420	8.35E-07	−0.817	*0.181*	*Pde11*	FBgn0085370	Syn.	Phosphodiesterase	Day *et al.* 2005
		2L.19068086	9.35E-07	−1.693	*0.036*	*CG10702*	FBgn0032752	Intron	Protein phosphorylation	Tweedie *et al.* 2009
						*CG17343*	FBgn0032751	Intron	Regulation of mitotic anaphase	Tweedie *et al.* 2009
		3L.12761401	1.40E-07	−1.800	*0.035*	*CG32113*	FBgn0052113	Syn.	Vesicle-mediated transport	Tweedie *et al.* 2009
		3R.22249607	4.98E-07	−1.144	*0.085*	*CG6503*	FBgn0040606	Down (813)	Unknown	NA
PC1	LGD	NA	NA	NA	NA	NA	NA	NA	NA	NA
PC2	LGD	2L.14109774	3.30E-07	1.122	0.225	*CG31769*	FBgn0051769	Syn.	Unknown	NA
		2R.6893862	6.94E-07	1.126	0.196	*luna*	FBgn0040765	Intron	DNA binding	Boutros *et al.* 2004
		X.12603566	9.74E-07	0.889	0.373	*Smr*	FBgn0263865	Intron	Regulation of mitotic cell cycle	Pile *et al.* 2002
PC3	LGD	3R.25503463	7.36E-07	0.884	0.302	*CAP-D2*	FBgn0039680	Intron	Mitotic sister chromatin segregation	Boutros *et al.* 2004
		3R.25504118	9.66E-07	1.009	0.269	*CAP-D2*	FBgn0039680	Syn.	Mitotic sister chromatin segregation	Boutros *et al.* 2004
PC4	LGD	2R.4176279	6.74E-07	1.640	0.042	NA	NA	NA	NA	NA
		3L.12262931	6.95E-07	0.666	0.442	*Pbgs*	FBgn0036271	3′ UTR	Porphobolinogin synthase	Golombieski *et al.* 2008
		3L.12412047	4.3E-07	2.33	0.021	NA	NA	NA	NA	NA
		3L.13348431	1.72E-07	1.682	0.042	*CG17687*	FBgn0036348	Down (391)	Unknown	NA
PC5	LGD	NA	NA	NA	NA	NA	NA	NA	NA	NA

Effect, effect size of minor allele; MAF, minor allele frequency. SNPs labeled NA are not within 1000 bp of an annotated gene. Lines with all NAs indicate no SNPs met significance threshold.

### Gene ontology analysis for enrichment

To determine whether the SNPs significantly associated with variation in each phenotype were clustered in genes with particular biological functions, we performed gene ontology (GO) enrichment analysis. Across all NIs and all diets, few categories were even nominally significant for enrichment and none was significant after correcting for multiple testing (Table 4). This may not be surprising because GO analysis of mapping results implicitly assumes the “infinitesimal model” of quantitative genetics, where many genes each contribute small but meaningful effects on the overall phenotype. We have no evidence that this is an appropriate model for our nutritional phenotypes, and we expect that, given the sample size of the DGRP, our experiment lacks power to identify SNPs of small effects.

**Table 4:** Gene Ontology term enrichment analysis for SNPs with *P* < 10^−4^

Index	Low-Glucose Diet		High-Glucose Diet		Both Diets Pooled	
Glucose	Apoptosis	(*P =* 0.016)	Signal transduction	(*P =* 0.020)	Lipid transport	(*P =* 0.030)
	DNA binding TF activity*^a^*	(*P =* 0.065)	Enzyme activator activity	(*P =* 0.039)	Protein folding	(*P* = 0.059)
	Catalytic activity	(*P =* 0.070)	G-protein-coupled receptor	(*P* = 0.068)		
	Plasma membrane	(*P* = 0.076)	Intracellular	(*P* = 0.075)		
			Receptor activity	(*P* = 0.079)		
Glycerol	Mitochondrion organization	(*P =* 0.044)	DNA packaging	(*P =* 0.052)	RNA binding	(*P =* 0.002)
	Ion transport	(*P =* 0.055)	Structural constituent of ribosome	(*P =* 0.052)	Translation	(*P =* 0.041)
	Transporter activity	(*P =* 0.056)	Ribosome	(*P =* 0.058)	Neurotransmitter transporter act.	(*P =* 0.065)
	Endopeptidase activity	(*P =* 0.084)	Plasma membrane	(*P =* 0.084)	Nucleo*^b^*	(*P =* 0.072)
	Transport	(*P =* 0.087)	Transport	(*P =* 0.089)	Behavior	(*P =* 0.077)
	Defense response	(*P =* 0.090)				
	Endocytosis	(*P =* 0.092)				
	Apoptosis	(*P =* 0.096)				
Glycogen	Structural constituent of cytoskeleton	(*P =* 0.051)	Multicellular organismal development	(*P =* 0.014)	Lipid metabolic process	(*P =* 0.030)
			Molecular function	(*P =* 0.095)	Lipid transport	(*P =* 0.087)
					Transport	(*P* = 0.099)
Mean wet weight	Protein kinase activity	(*P =* 0.010)	Intracellular	(*P =* 0.005)	Cytoskeleton organization	(*P =* 0.032)
	Protein modification process	(*P =* 0.025)	Cytoskeleton organization	(*P =* 0.009)	Extracellular region	(*P =* 0.035)
	Response to stress	(*P =* 0.031)	Organelle development	(*P =* 0.013)		
	RNA binding	(*P =* 0.036)	Endopeptidase activity	(*P =* 0.033)		
	Cytosol	(*P =* 0.054)	Protein kinase activity	(*P =* 0.039)		
	Intracellular protein transport	(*P =* 0.086)	Peptidase activity	(*P =* 0.049)		
	Transcription factor binding	(*P =* 0.089)	Proteolysis	(*P =* 0.033)		
			RNA binding	(*P =* 0.072)		
			Cytoskeleton	(*P =* 0.079)		
			Protein modification process	(*P =* 0.095)		
			DNA-dependent transcription	(*P =* 0.096)		
Protein	Extracellular region	(*P =* 0.008)	Cell death	(*P =* 0.010)	Centrosome	(*P =* 0.005)
			Intracellular	(*P =* 0.049)	Sensory perception	(*P =* 0.032)
			DNA binding	(*P =* 0.056)	Cytoskeleton organization	(*P* = 0.058)
			Extracellular region	(*P =* 0.061)	Neurotransmitter transporter activity	(*P* = 0.099)
			Molecular function	(*P =* 0.079)		
Triglyceride	Motor activity	(*P =* 0.029)	DNA binding TF activity*^a^*	(*P =* 0.012)	Transporter activity	(*P =* 0.005)
	Cell death	(*P =* 0.033)	Nucleus	(*P =* 0.024)	Centrosome	(*P =* 0.016)
	Cell communication	(*P =* 0.043)	Nucleic acid binding	(*P =* 0.078)	Cellular component	(*P =* 0.017)
			Intracellular	(*P =* 0.091)	DNA binding TF activity*^a^*	(*P =* 0.025)
					Transport	(*P =* 0.026)
					Ion transport	(*P =* 0.079)
					DNA binding	(*P =* 0.091)

a “DNA binding TF activity” is “sequence-specific DNA binding transcription factor activity.”

b “Nucleo” is “nucleobase, nucleoside, nucleotide, and nucleic acid metabolic process.

## Discussion

We found significant genetic variation for wet weight as well as five nutritional indices (levels of glycogen, free glucose, soluble protein, triglycerides, and free glycerol) in the DGRP after rearing on two different diets that varied in glucose content. Several of these nutritional indices and the principal components describing them jointly are correlated with phenotypes that have been measured by other researchers. Because the complete genomes have been sequenced for all of the lines in the DGRP, we could conduct genome-wide association mapping to identify candidate genes that may influence *Drosophila* metabolic status in response to diet.

We were able to identify genetic correlations among the traits we measured and between our traits and phenotypes measured by independent groups in other studies. Many of these correlations make good biological sense. For example, starvation stress resistance is positively correlated with wet weight and with stores of glucose and glycogen, consistent with a simple interpretation that genotypes that store more nutrients are more resistant to starvation. The correlations among other phenotypes were less intuitive but may motivate follow-up examination. For example, we found correlations between endoplasmic reticulum stress and several nutritional indices (glycogen, glycerol, triglycerides), suggesting that nutrients play a role in modulating the ER stress response. One concern could be that spurious correlations arise due to variable inbreeding depression among the lines. However, we do not believe this would be a sufficient explanation because at least some of the correlations appear to be negatively correlated with respect to fitness. For example, wet weight was negatively correlated with male aggression (*P =* 0.044), where we would presume that both greater wet weight and more aggressive males would be more “fit.” However, guessing at the fitness value of nutritional indices is obviously difficult. For example, we simply do not know *a priori* whether flies with more glycogen stores are inherently more or less fit than flies storing less glycogen, and the answer probably depends on the environmental conditions.

Our genome-wide association mapping implicated many genes as explaining natural variation for nutritional phenotypes, and these can be targeted for more thorough follow-up study. One striking pattern is the over-representation of genes involved in nervous system development and behavior. This may be an artifact of the observation that neurological genes tend to be large and therefore provide a larger target for association studies (Mackay *et al.* 2012; Chow *et al.* 2013a). Neurological terms were generally not enriched in our GO analysis that controlled for gene size. A majority of significantly associated SNPs were intronic, suggesting that gene expression variation may play a major role in determining variability in nutritional phenotypes. Generally speaking, the mapping results presented here can provide a starting point for further research on these important traits.

## Supplementary Material

Supporting Information
