# Supplementary material for: A Genome-Wide Association Study for Nutritional Indices in Drosophila
Source: G3 (Bethesda). 2015 Jan 12;5(3):417–25. doi: 10.1534/g3.114.016477 (PMC4349095; doi:10.1534/g3.114.016477)
Supplement: Supporting Information [file supp_5_3_417__index.html]

A Genome-Wide Association Study for Nutritional Indices in Drosophila — Supporting Information 

# A Genome-Wide Association Study for Nutritional Indices in *Drosophila*

## Supporting Information for Unckless, Rottschaefer, and Lazzaro, 2015

**Files in this Data Supplement:**

- Supporting Information - Tables S1-S3 and Figure S1 (PDF, 587 KB)
- Table S1 - (A) F value (*P*-value) from ANOVA determining which experimental factors (row) predict each phenotype columns. (B) Effect of genetic line in determining traits on each diet: Z-values (*P*-values). (PDF, 134 KB)
- Table S3 - Loadings of principal component analysis of nutritional phenotype line means. (PDF, 104 KB)
- Figure S1 - Quantile-quantile plots for genome-wide association *P*-values for measured phenotypes, where HGD is "high glucose diet" and LGD is "low glucose diet." (PDF, 554 KB)
- Table S2 - Line mean estimates for nutritional phenotypes. (.xlsx, 79 KB)
