# Supplementary material for: A Genome-Wide Association Study for Nutritional Indices in Drosophila
Source: G3 (Bethesda). 2015 Jan 12;5(3):417–25. doi: 10.1534/g3.114.016477 (PMC4349095; doi:10.1534/g3.114.016477)
Supplement: Supporting Information [file supp_g3.114.016477_016477SI.pdf]

G3: Genes | Genomes | Genetics

A genome-wide association study for nutritional indices in *Drosophila*

Robert L. Unckless[*,1], Susan M. Rottschaefer[*] and Brian P. Lazzaro[*]

*Department of Entomology, Cornell University, Ithaca, NY 14853

[1]Corresponding author: E-mail: rlu1@cornell.edu

DOI: 10.1534/g3.114.016477

Table S1a F value (*P*-value) from ANOVA determining which experimental factors (row) predict each phenotype columns

Factor	Glucose	Protein	Triglyceride	Glycerol	Glycogen	Wet weight
Wolbachia status	1.36 (0.24)	6.25 (0.013)	0.01 (0.92)	0.58 (0.45)	0.35 (0.56)	1.11 (0.29)
Diet	29.86 (<0.0001)	4.06 (0.044)	0.25 (0.62)	18.42 (<0.0001)	0.56 (0.45)	50.24 (<0.0001)
Line(*Wolbachia*)	1.74 (<0.0001)	1.39 (0.004)	1.38 (0.0049)	2.30 (<0.0001)	3.61 (<0.0001)	7.47 (<0.0001)
Diet x Line(*Wolb*)	1.16 (0.12)	0.81 (0.94)	1.03 (0.41)	1.05 (0.35)	1.04 (0.36)	1.33 (0.012)
Block(Diet)	9.47 (<0.0001)	8.60 (<0.0001)	5.04 <0.0001)	31.52 (<0.0001)	15.65 (<0.0001)	16.35 (<0.0001)

Table S1b Effect of genetic line in determining traits on each diet: Z-values (*P*-values).

Diet	Glucose	Protein	Triglyceride	Glycerol	Glycogen	Wet weight
Low glucose	1.44 (0.0041)	0.97 (0.56)	1.35 (0.017)	1.65 (0.0001)	2.21 (<0.0001)	4.08 (<0.0001)
High glucose	1.46 (0.0038)	1.26 (0.051)	0.99 (0.53)	1.68 (0.0001)	2.48 (<0.0001)	4.96 (<0.0001)

Table S2

Line mean estimates for nutritional phenotypes

Table S2 is available for download at http://www.g3journal.org/lookup/suppl/doi:10.1534/g3.114.016477/-/DC1

Table S3   Loadings of principal component analysis of nutritional phenotype line means

Treatment	Component	PC1	PC2	PC3	PC4	PC5
Low glucose	St. dev.	1.25	1.10	0.99	0.82	0.76
	Variance prop.	0.31	0.24	0.20	0.13	0.12
	Protein	-0.22	-0.42	-0.79	-0.38	-0.10
	Glucose	-0.59	0.32	-0.15	0.12	0.71
	Triglyceride	0.37	0.44	-0.59	0.56	-0.11
	Glycerol	-0.56	-0.33	0.08	0.63	-0.40
	Glycogen	-0.38	0.65	0.002	-0.36	-0.55
High glucose	St. dev.	1.21	1.07	1.02	0.87	0.78
	Variance prop.	0.29	0.23	0.21	0.15	0.12
	Protein	-0.04	0.77	0.26	-0.55	-0.19
	Glucose	0.65	-0.23	0.06	-0.10	-0.71
	Triglyceride	0.35	0.18	0.73	0.49	0.25
	Glycerol	0.22	0.56	-0.57	0.55	-0.11
	Glycogen	0.64	-0.005	-0.25	-0.38	0.62

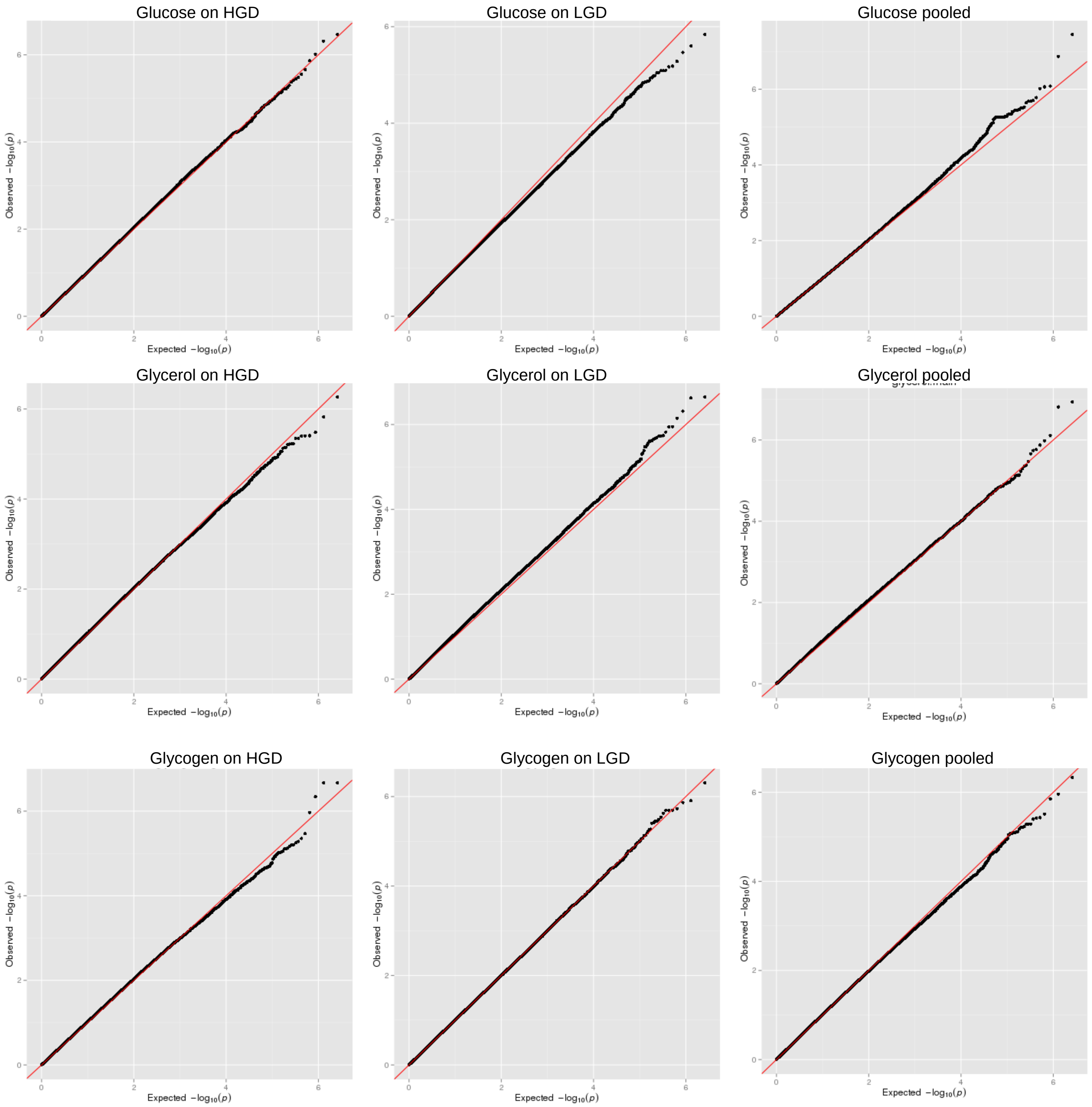
Glucose on HGD
Glucose on LGD
Glucose pooled
Glycerol on HGD
Glycerol on LGD
Glycerol pooled
Glycogen on HGD
Glycogen on LGD
Glycogen pooled
Observed $-\log_{10}(p)$
Expected $-\log_{10}(p)$

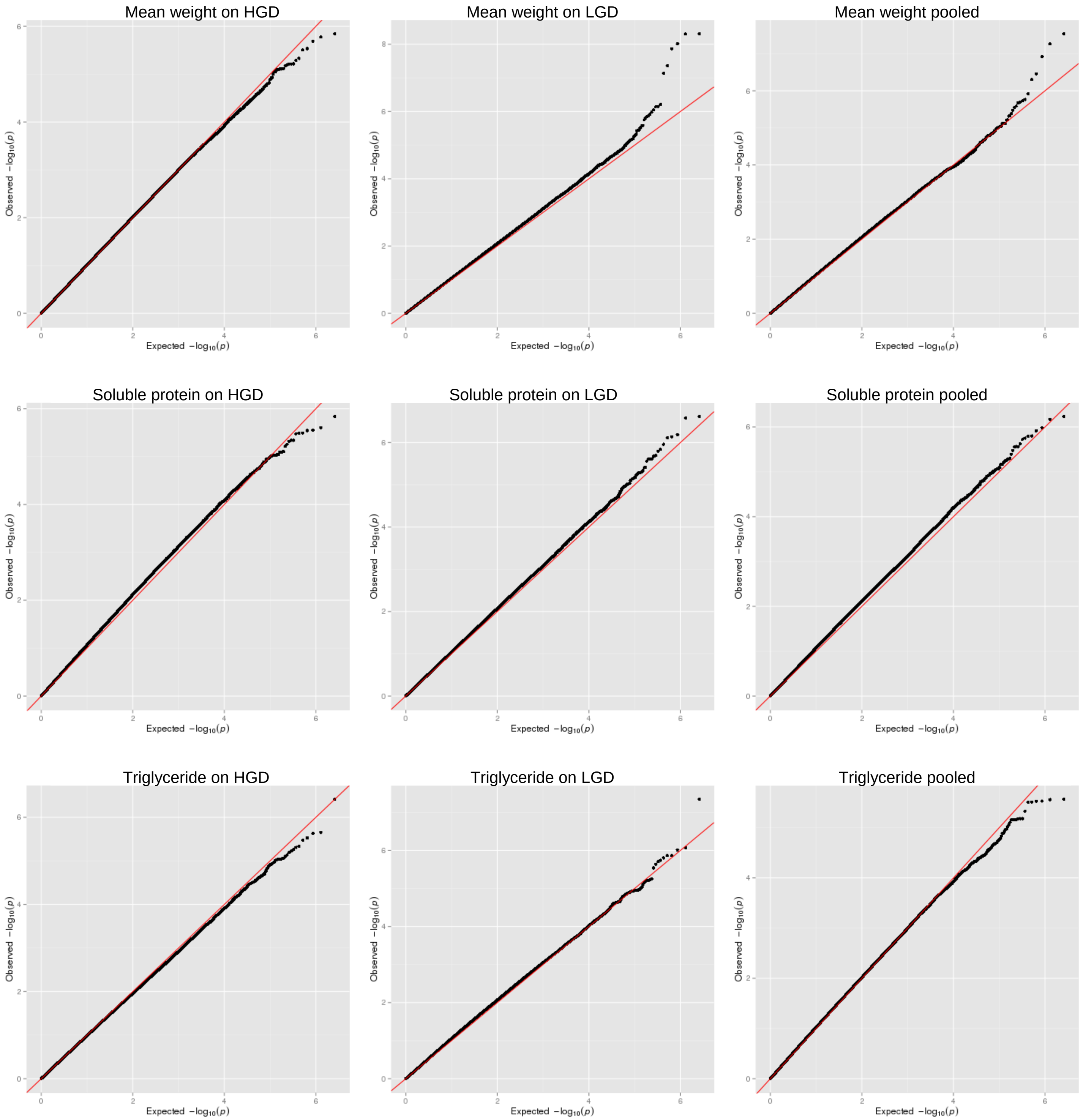
Mean weight on HGD
Mean weight on LGD
Mean weight pooled
Soluble protein on HGD
Soluble protein on LGD
Soluble protein pooled
Triglyceride on HGD
Triglyceride on LGD
Triglyceride pooled
Observed $-\log_{10}(p)$
Expected $-\log_{10}(p)$

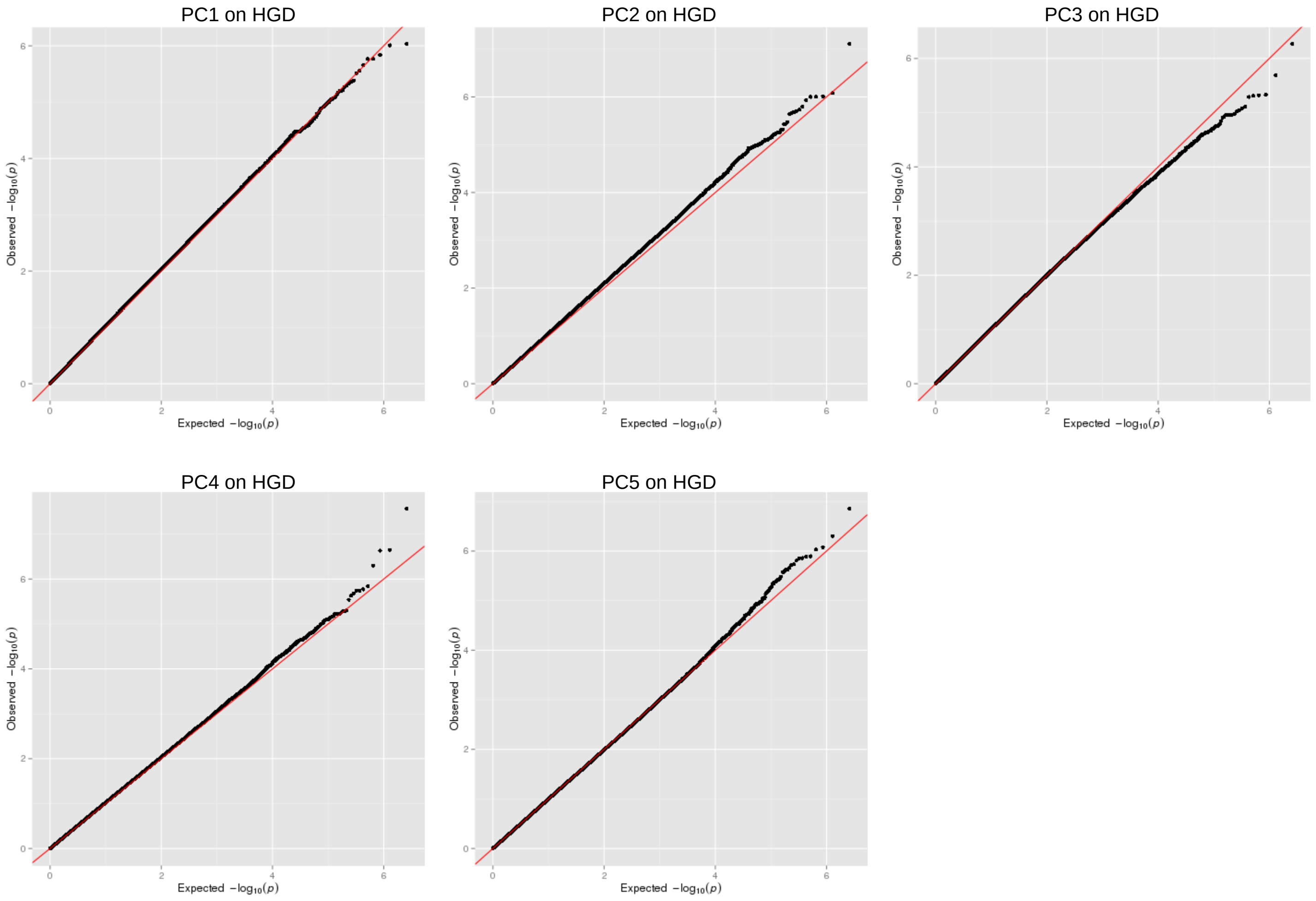
PC1 on HGD
PC2 on HGD
PC3 on HGD
PC4 on HGD
PC5 on HGD
Observed $-\log_{10}(p)$
Expected $-\log_{10}(p)$

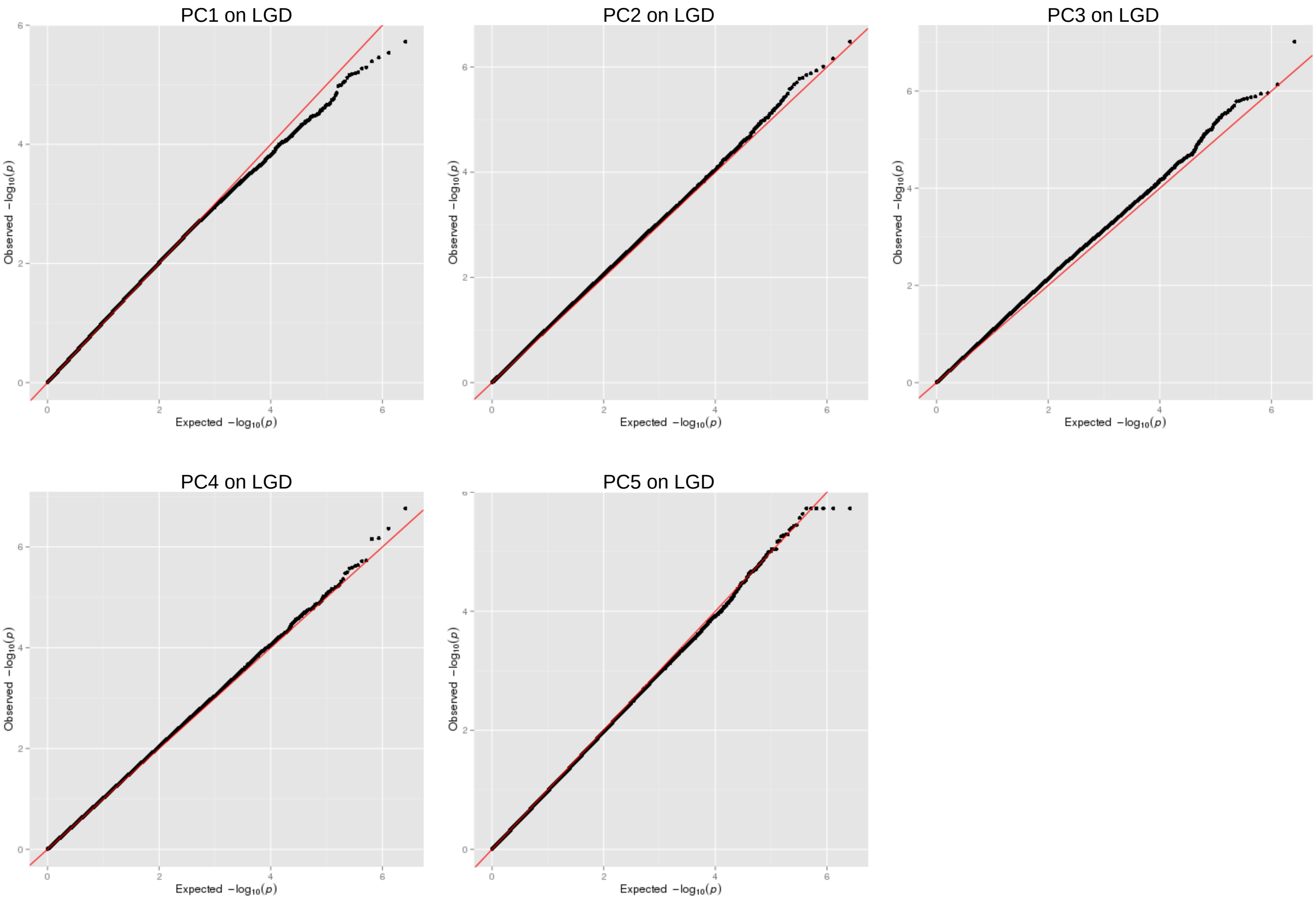

Figure S1 Quantile-quantile plots for genome-wide association *P*-values for measured phenotypes, where HGD is "high glucose diet" and LGD is "low glucose diet"
