# Supplementary material for: A Genome-Wide Association Study for Nutritional Indices in Drosophila
Source: G3 (Bethesda). 2015 Jan 12;5(3):417–25. doi: 10.1534/g3.114.016477 (PMC4349095; doi:10.1534/g3.114.016477)
Supplement: Supporting Information [file supp_g3.114.016477_TableS1.pdf]

Table S1a F value (*P*-value) from ANOVA determining which experimental factors (row) predict each phenotype columns

Factor	Glucose	Protein	Triglyceride	Glycerol	Glycogen	Wet weight
Wolbachia status	1.36 (0.24)	6.25 (0.013)	0.01 (0.92)	0.58 (0.45)	0.35 (0.56)	1.11 (0.29)
Diet	29.86 (<0.0001)	4.06 (0.044)	0.25 (0.62)	18.42 (<0.0001)	0.56 (0.45)	50.24 (<0.0001)
Line(*Wolbachia*)	1.74 (<0.0001)	1.39 (0.004)	1.38 (0.0049)	2.30 (<0.0001)	3.61 (<0.0001)	7.47 (<0.0001)
Diet x Line(*Wolb*)	1.16 (0.12)	0.81 (0.94)	1.03 (0.41)	1.05 (0.35)	1.04 (0.36)	1.33 (0.012)
Block(Diet)	9.47 (<0.0001)	8.60 (<0.0001)	5.04 <0.0001)	31.52 (<0.0001)	15.65 (<0.0001)	16.35 (<0.0001)

Table S1b Effect of genetic line in determining traits on each diet: Z-values (*P*-values).

Diet	Glucose	Protein	Triglyceride	Glycerol	Glycogen	Wet weight
Low glucose	1.44 (0.0041)	0.97 (0.56)	1.35 (0.017)	1.65 (0.0001)	2.21 (<0.0001)	4.08 (<0.0001)
High glucose	1.46 (0.0038)	1.26 (0.051)	0.99 (0.53)	1.68 (0.0001)	2.48 (<0.0001)	4.96 (<0.0001)
