# Supplementary material for: A Genome-Wide Association Study for Nutritional Indices in Drosophila
Source: G3 (Bethesda). 2015 Jan 12;5(3):417–25. doi: 10.1534/g3.114.016477 (PMC4349095; doi:10.1534/g3.114.016477)
Supplement: Supporting Information [file supp_g3.114.016477_TableS3.pdf]

Table S3 Loadings of principal component analysis of nutritional phenotype line means

Treatment	Component	PC1	PC2	PC3	PC4	PC5
Low glucose	St. dev.	1.25	1.10	0.99	0.82	0.76
	Variance prop.	0.31	0.24	0.20	0.13	0.12
	Protein	-0.22	-0.42	-0.79	-0.38	-0.10
	Glucose	-0.59	0.32	-0.15	0.12	0.71
	Triglyceride	0.37	0.44	-0.59	0.56	-0.11
	Glycerol	-0.56	-0.33	0.08	0.63	-0.40
	Glycogen	-0.38	0.65	0.002	-0.36	-0.55
High glucose	St. dev.	1.21	1.07	1.02	0.87	0.78
	Variance prop.	0.29	0.23	0.21	0.15	0.12
	Protein	-0.04	0.77	0.26	-0.55	-0.19
	Glucose	0.65	-0.23	0.06	-0.10	-0.71
	Triglyceride	0.35	0.18	0.73	0.49	0.25
	Glycerol	0.22	0.56	-0.57	0.55	-0.11
	Glycogen	0.64	-0.005	-0.25	-0.38	0.62
