# Supplementary material for: A Genome-Wide Association Study for Nutritional Indices in Drosophila
Source: G3 (Bethesda). 2015 Jan 12;5(3):417–25. doi: 10.1534/g3.114.016477 (PMC4349095; doi:10.1534/g3.114.016477)
Supplement: Supporting Information [file supp_g3.114.016477_FigureS1.pdf]

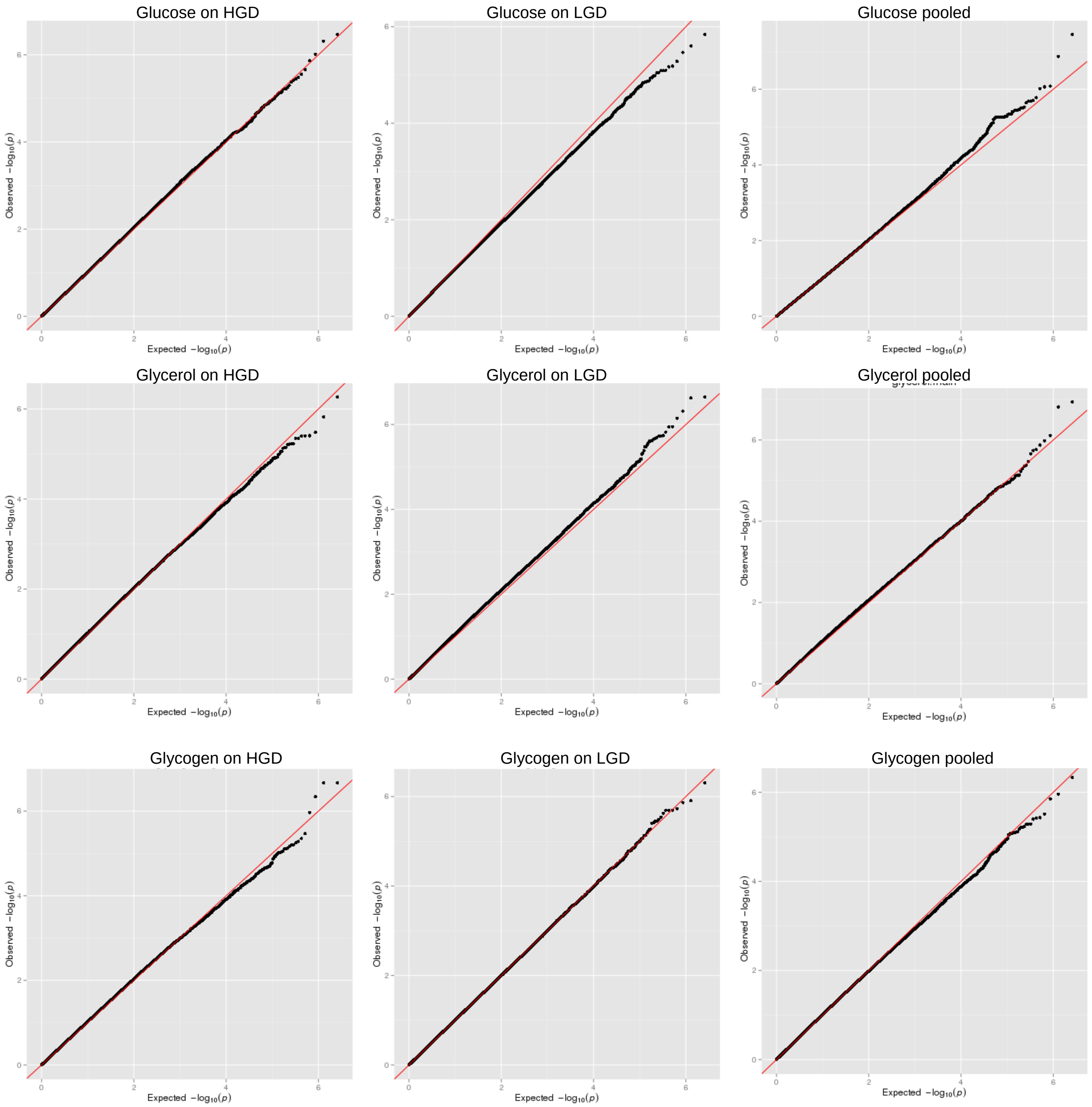
Glucose on HGD
Glucose on LGD
Glucose pooled
Glycerol on HGD
Glycerol on LGD
Glycerol pooled
Glycogen on HGD
Glycogen on LGD
Glycogen pooled
Observed $-\log_{10}(p)$
Expected $-\log_{10}(p)$

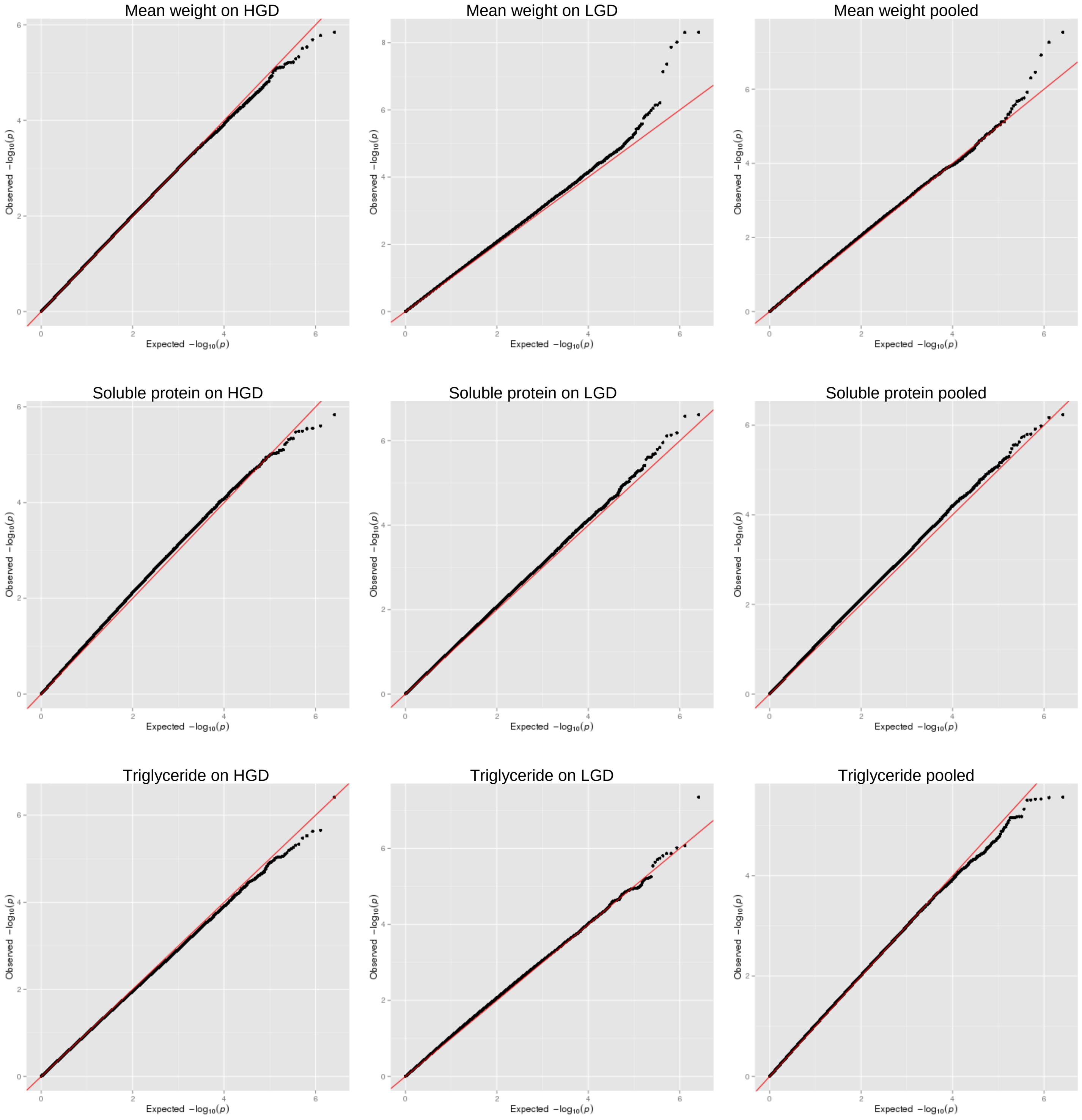
Mean weight on HGD
Mean weight on LGD
Mean weight pooled
Soluble protein on HGD
Soluble protein on LGD
Soluble protein pooled
Triglyceride on HGD
Triglyceride on LGD
Triglyceride pooled
Observed $-\log_{10}(p)$
Expected $-\log_{10}(p)$

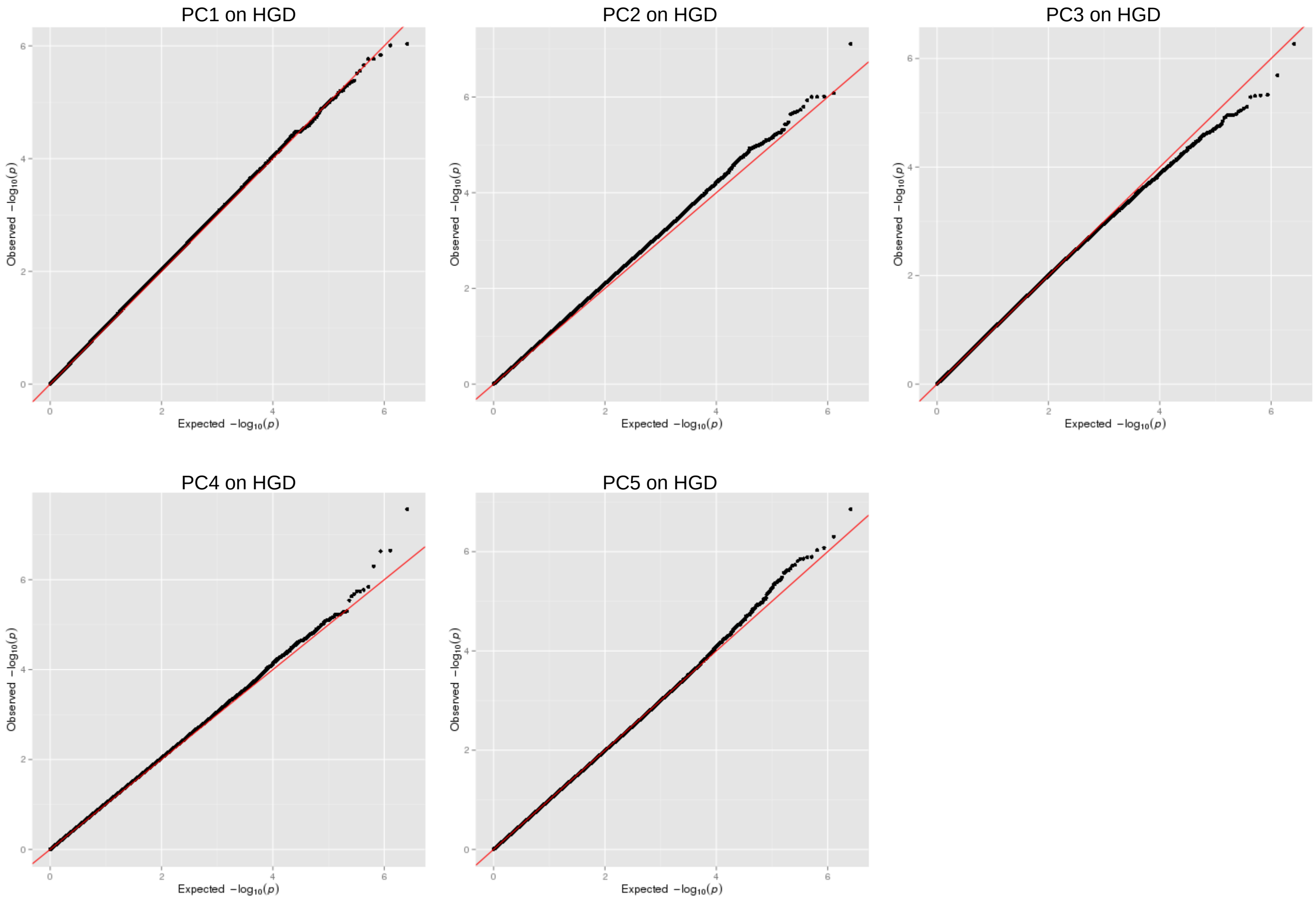
PC1 on HGD
PC2 on HGD
PC3 on HGD
PC4 on HGD
PC5 on HGD
Observed $-\log_{10}(p)$
Expected $-\log_{10}(p)$

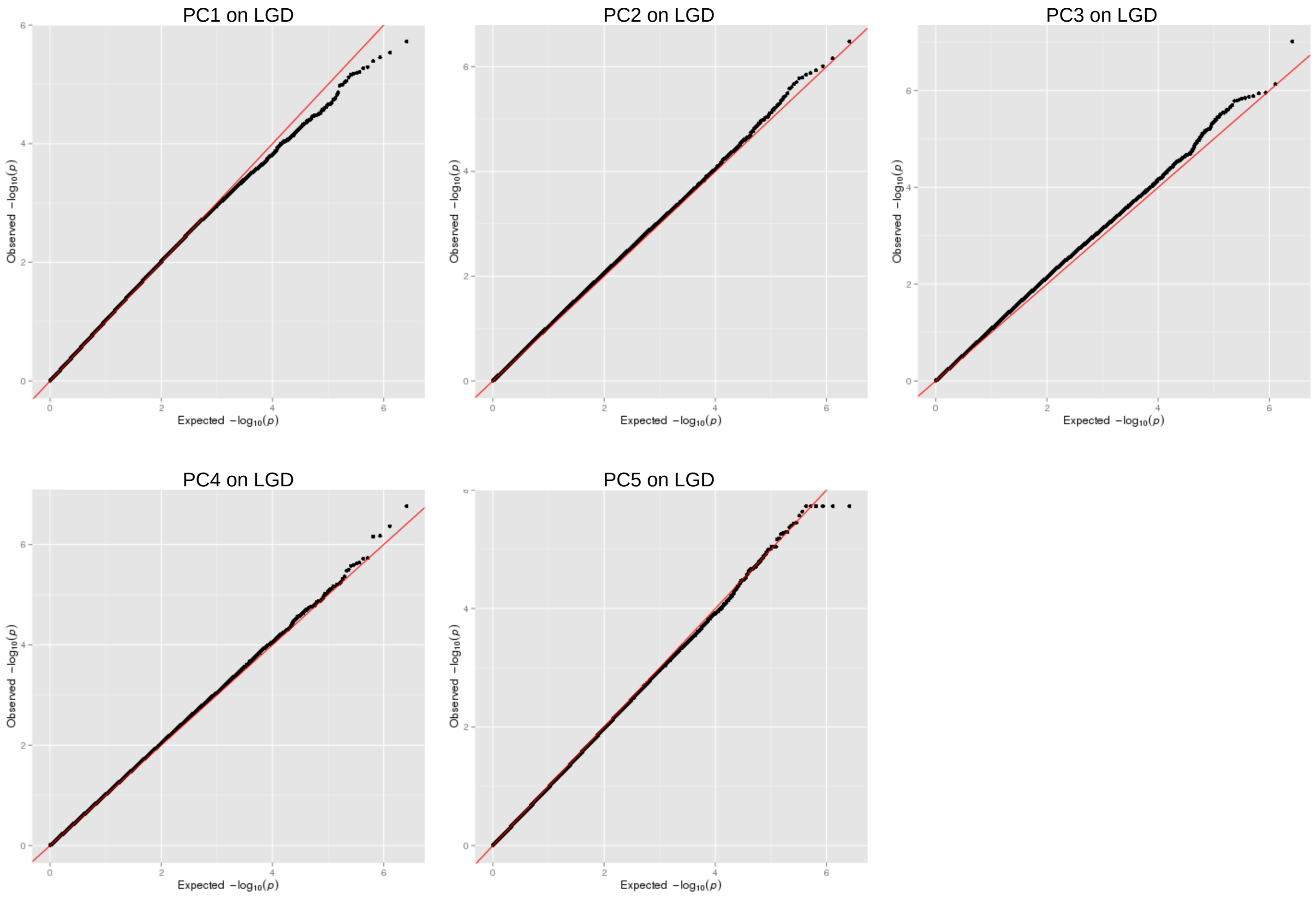

Figure S1 Quantile-quantile plots for genome-wide association *P*-values for measured phenotypes, where HGD is “high glucose diet” and LGD is “low glucose diet”
